# Latent profiles of social connectedness and associated factors in maintenance hemodialysis patients

**DOI:** 10.1038/s41598-026-59651-z

**Published:** 2026-06-24

**Authors:** Yun Zhang, Zhiyan Sun, Naiyue Ye, Xun Zhou, Menghan Zheng, Peili Xu

**Affiliations:** 1 School of Nursing, Anhui University of Chinese Medicine, Hefei, Anhui Province 230012 China; 2 Hemodialysis Unit, The First People’s Hospital of Hefei, Hefei, Anhui Province 231220 China; 3 School of Nursing, Wannan Medical University, Wuhu, 241002 China; 4 Nursing Department, The First People’s Hospital of Hefei, Hefei, Anhui Province 231220 China

**Keywords:** Maintenance hemodialysis, Social connectedness, Latent profile analysis, Social ecosystem theory, Health care, Nephrology, Risk factors

## Abstract

To date, no study has characterized the heterogeneity of social connectedness among patients undergoing maintenance hemodialysis (MHD). This study used latent profile analysis (LPA) to identify distinct latent profiles of social connectedness in this population and to explore the factors associated with different profiles. This cross-sectional study enrolled 365 patients undergoing MHD at a tertiary hospital in Anhui Province, China, from September 2025 to March 2026. Data were collected using a demographic questionnaire, the Social Connectedness Scale-Revised (SCS-R), the Social Support Rating Scale (SSRS), and the 10-item Connor-Davidson Resilience Scale (CD-RISC-10). Data analysis was performed using SPSS 26.0 and Mplus 8.3. A total of 345 valid questionnaires were collected. The response rate was 94.52% (345/365). We identified three potential profile categories: low social connectedness (*n* = 110, 31.9%), moderate social connectedness (*n* = 146, 42.3%), and high social connectedness (*n* = 89, 25.8%). Multivariate logistic regression analysis identified educational level, employment status, dialysis shift, types of chronic diseases, resilience, and social support as factors significantly associated with these three profiles (*P* < 0.05). The findings indicate that social connectedness among MHD patients exhibits significant heterogeneity across subgroups and is associated with a variety of factors. Healthcare professionals should identify patients’ social connectedness profiles early and implement targeted interventions to improve their social connectedness.

**Supplementary Information:**

The online version contains supplementary material available at 10.1038/s41598-026-59651-z.

## Introduction

There are approximately 132 million people in China living with chronic kidney disease, of whom around 2% progress to end-stage renal disease (ESRD) each year^1^. It is projected that by 2050, chronic kidney disease will become the fifth leading cause of death worldwide^2^. Dialysis remains the primary treatment option for patients with ESRD^3^, and there are currently around 916,000 patients in China receiving dialysis treatment^4^. Although dialysis can effectively prolong survival, patients often face multiple challenges as the disease progresses. These include declining physical function, increasing complications, and a growing financial burden^5^. Such factors not only reduce quality of life but also weaken patients’ social participation and their level of social functioning. Ultimately, this leads to poor social connectedness.

Social connectedness refers to an individual’s subjective perception of their close relationships^6^ and is widely regarded as a key determinant of mental health. When individuals experience high levels of social connectedness, they tend to feel close to others, perceive them as friendly and approachable, and actively participate in social activities while integrating into social groups^7^. Research suggests^8^ that social connections are associated with increased life expectancy, improved cognitive function, and enhanced neuroendocrine and immune regulation. Conversely, a decline in social connections can lead to depression, loneliness and anxiety, and may even increase the risk of suicide^9^. Patients with MHD are more likely to find themselves in a disadvantaged social position within their peer group, long-term treatment and frequent dialysis tend to fragment their social time and cause their social networks to gradually shrink. Furthermore, the stigma associated with the disease often exacerbates patients’ sense of social isolation^10^, accompanied by feelings of inferiority and neglect, which further undermines their positive social identity. It is therefore necessary to investigate their social connectedness, as this is of great practical importance for promoting mental health and restoring social functioning.

Currently, research on social connectedness has largely focused on the field of psychology^11,12^ with few studies investigating patients with MHD. Furthermore, most existing studies treat participants as a homogeneous group, conducting cross-sectional analyses based on total scale scores, thus ignoring inter-individual heterogeneity. Latent Profile Analysis (LPA)^13^ is an individual-centred approach. Its advantage is that it can identify latent profiles and distinguish distinct subgroups, providing a basis for targeted interventions. This method has been widely applied in research on chronic diseases and psychological outcomes^14,15^.

According to the social–ecological systems theory refined by Charles Zastrow^16^, individual development and adaptation are the joint outcomes of interactions among the micro-, meso-, and macro-systems. This theory emphasises the dynamic interaction between the individual and the social environment. This multi-level analytical perspective is particularly well-suited to revealing the complexity and multi-dimensional origins of social connectedness. We regard social connectedness as the core adaptive behaviour for MHD patients. Psychological resilience as a core individual factor at the microsystem level, social support as an environmental factor at the mesosystem level, and place of residence and medical insurance type as institutional factors at the macrosystem level are key variables associated with social connectedness. Psychological resilience, as a vital internal protective resource for individuals, refers to the ability to maintain or restore mental and physical health in the face of adversity. It has been shown to be associated with improved treatment adherence, reduced psychological distress and enhanced long-term recovery^17^; higher levels of psychological resilience can increase well-being and life satisfaction, whilst reducing symptoms of anxiety and depression^18,19^, thereby may strengthen patients’ willingness to actively maintain interpersonal connections and participate in social activities.

Social support is a key protective factor, encompassing emotional, material and informational support from family, friends and healthcare professionals; it serves as a vital means of maintaining social connections for MHD patients. Previous studies have reported^20^ that adequate social support can improve the quality of life and treatment adherence among dialysis patients, alleviate their suffering, and promote their social participation. Social support not only has a direct impact on an individual’s mental health but also exerts a protective effect indirectly by enhancing psychological resilience^21^. Place of residence and medical insurance type are external factors shaping patients’ daily lives, resource access and behavioural choices. Residential conditions are linked to patients’ community engagement and healthcare access. Rural patients generally lack social resources and suffer from poor transport links^22,23^, restricting their ability to build and sustain social connectedness. Medical insurance coverage is closely linked to financial burden and social security. Patients with low reimbursement or self-funded dialysis experience financial stress, develop negative emotions and deliberately avoid social activities^24,25^, further weakening their social connectedness.

**Fig. 1:**
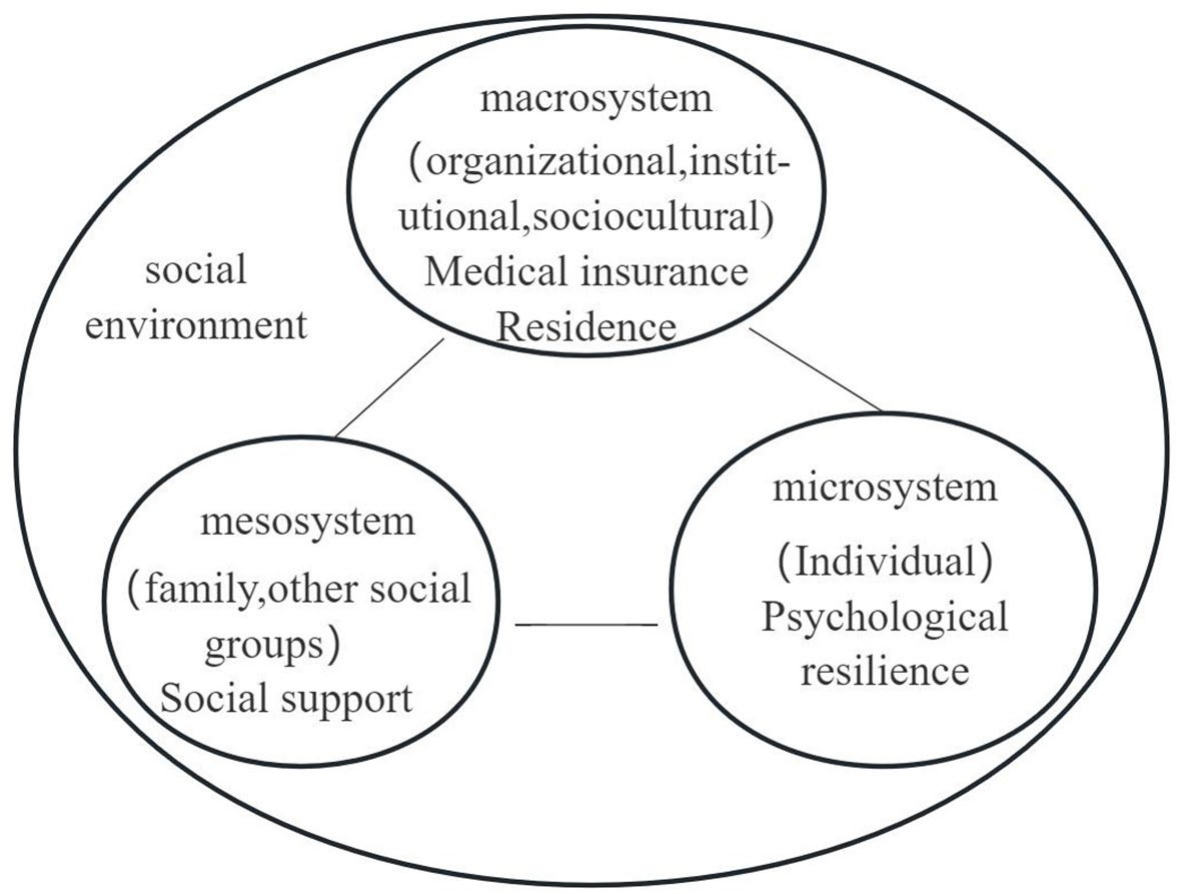
Theoretical hypothesis framework.

Currently, research examining how the microsystem (psychological resilience), mesosystem (social support), and macrosystem (medical insurance, place of residence, etc.) collectively relate to the heterogeneous distribution of social connectedness among MHD patients remains relatively scarce, as shown in Fig. 1.Traditional single-dimensional analyses are insufficient to capture this complexity. Conducting research from an integrated multi-system perspective can provide key theoretical foundations for individualized stratified care and offer new insights for patient management. Accordingly, this study applies the social-ecological framework and latent profile analysis to identify subgroups of social connectedness in MHD patients and examine relevant factors. It aims to address the limitations of existing research, inform targeted and stratified clinical interventions, and ultimately improve patients’ social functioning and overall well-being.

## Methods

### Survey subjects

A convenience sampling method was employed to select patients undergoing maintenance hemodialysis (MHD) at a tertiary comprehensive hospital in Hefei, China, from September 2025 to March 2026. Inclusion criteria: (1) Diagnosed with end-stage renal disease (ESRD)^26^, regular dialysis for ≥ 3 months; (2) Age ≥ 18 years; (3) Clear consciousness and normal communication abilities. Exclusion criteria: (1) Mental illness or cognitive impairment; (2) Acute infections or critical conditions. Based on the principles of logistic regression^27^, the sample size should be at least 10 times the number of independent variables. As this study included 22 independent variables, and accounting for a 10%-20% nonresponse rate, the estimated sample size ranged from 245 to 275 participants. In line with previous research on latent profile analysis, a sample size of at least 300 is recommended^28^. The final sample size consisted of 365 participants. This research was approved by the Ethics Committee of Hefei First People’s Hospital. (No.2025-177-01).

### Research instruments

#### Demographic questionnaire

Based on a review of the literature and the objectives of this study, a comprehensive questionnaire was designed. This included sociodemographic data such as the patients’ gender, age, marital status, educational level, employment status, place of residence and type of medical insurance, as well as disease-related data such as primary disease, duration of dialysis, frequency of dialysis, type of chronic conditions, number of medications taken, level of self-care ability and self-assessed health status.

#### Social Connectedness Scale-Revised (SCS-R)

The SCS-R was developed by Lee et al.^6^. In 2022, Chinese scholar Wu Caizhi adapted it into Chinese for use in assessing respondents’ social connectedness^7^. The scale comprises nine items. Each item is rated on a 6-point Likert scale (1–6), with total scores ranging from 9 to 54; a higher score indicates a higher level of social connectedness. In our study, the scale’s Cronbach’s α was 0.923, the split-half reliability was 0.922, the *KMO* measure was 0.922, and Bartlett’s test of sphericity showed χ^2^ = 821.516 (*P* < 0.001).

#### 10-item Connor-Davidson Resilience Scale (CD-RISC-10)

This scale was developed by Campbell-Sills et al.^29^ to assess an individual’s level of psychological resilience. This study used the Chinese version translated by Wang^30^, which consists of 10 items. All items are rated on a 5-point Likert scale (0–4), with total scores ranging from 0 to 40; higher scores indicate a higher level of psychological resilience. In this study, Cronbach’s α for the patients was 0.842.

#### Social Support Rating Scale (SSRS)

The SSRS, developed by Xiao Shuiyuan^31^, was used to assess participants’ social support. This scale consists of 10 items across three dimensions: objective support (3 items), subjective support (4 items), and support utilization (3 items). Items 1–4 and 8–10 use a 4-point Likert scale. For item 5, responses ranging from “none” to “full support” were scored from 1 to 4. For items 6 and 7, the score is calculated based on the number of support sources reported. Total scores range from 12 to 66, with higher scores indicating greater levels of social support. In this study, Cronbach’s α for the patients was 0.807.

### Data collection

Prior to the survey, all investigators received standardised training. Following the obtaining of informed consent from patients, they were asked to complete the questionnaire anonymously. Furthermore, the participants were informed of the study purpose, procedures and instructions for questionnaire completion. Once completed, the questionnaires were collected on the spot and checked for any missing or incomplete entries to ensure their completeness. Throughout the study, we conducted regular quality control checks to ensure the reliability and authenticity of the data collected. Following the collection of the questionnaires, those containing logical errors or patterns of unreasonable responses were excluded. A total of 365 questionnaires were distributed, with 345 valid responses collected, yielding an effective response rate of 94.52%.

### Statistical methods

Latent profile analysis (LPA) was conducted using Mplus 8.3, with the scores of the 9 items on the social connectedness scale serving as external indicators. The model fit evaluation metrics included^32^ Akaike information criterion (AIC), Bayesian information criterion (BIC), and adjusted Bayesian information criterion (aBIC)—lower values indicate better model fit. Entropy values (> 0.8 indicates good classification accuracy); the Lo-Mendell-Rubin likelihood ratio test (LMRT), and bootstrapped likelihood ratio test (BLRT), with significance (*P* < 0.05) indicating that the k class model fitted the data better than the k-1 class model. Data analysis was performed using SPSS 26.0. Quantitative data with a normal distribution were described as the mean and standard deviation; while the skewed data were expressed as median and interquartile range; and categorical data were described as frequencies and percentages. Intergroup comparisons were performed using the chi-square test or the Kruskal-Wallis *H* test. Multivariate logistic regression was employed to identify factors associated with different categories of social connectedness, with *P <* 0.05 considered statistically significant. The optimal model was determined through a comprehensive analysis of the fit indices, combined with clinical judgment.

## Results

### General characteristics of survey subjects

Among the 345 MHD patients, 211 were male (61.20%), and 134 were female (38.80%). The causes of dialysis were as follows: 124 patients (35.90%) had chronic nephritis, 87 (25.20%) had hypertensive nephropathy, 84 (24.30%) had diabetic nephropathy, and 50 (14.50%) had other conditions. Details are shown in Table 1.

**Table 1:** General information of study subjects (*n* = 345).

Variable	Number(%)	Variable	Number(%)
Gender		Weekly dialysis duration (h)	
Male	211 (61.20)	< 12	37 (10.70)
Female	134 (38.80)	≥ 12	308 (89.30)
Age (years)		Dialysis vintage	
18–44	49 (14.20)	< 1 year	51 (14.80)
45–59	115 (33.30)	1–5 years	161 (46.70)
≥ 60	181 (52.50)	> 5 years	133 (38.60)
Marital status		Types of chronic diseases	
Married	277 (80.30)	2	135 (39.10)
Unmarried	18 (5.20)	3	157 (45.50)
Divorced/Widowed	50 (14.50)	≥ 4	53 (15.40)
Place of residence		Number of medications (types)	
Rural	78 (22.60)	< 3	205 (59.40)
Urban	267 (77.40)	≥ 3	140 (40.60)
Education level		Primary disease	
Junior high school or lower	130 (37.70)	Chronic glomerulonephritis	124 (35.90)
High school or vocational school	125 (36.20)	Hypertensive nephropathy	87(25.20)
College degree or above	90 (26.10)	Diabetic nephropathy	84 (24.30)
Employment status		Others	50 (14.50)
Currently employed	68 (19.70)	Dialysis shift	
Leaving employment/ Unemployed	193 (55.90)	Morning dialysis	147 (42.60)
Retirement	84 (24.30)	Afternoon dialysis	159 (46.10)
Monthly per capita household income (CNY, yuan)		Evening dialysis	39 (11.30)
< 3000	143 (41.40)	Activities of daily living	
3000–6000	145 (42.00)	Independent	260 (75.40)
> 6000	57 (16.50)	Partially dependent	72 (20.90)
insurance		Completely dependent	13 (3.80)
Employee medical insurance	150 (43.50)	Self-rated health status	
New Rural Cooperative Medical Scheme	52 (15.10)	Poor	89 (25.80)
Resident Medical Insurance	143 (41.40)	Fair	116 (33.60)
Dialysis frequency		Good	140 (40.60)
< 3 times a week	23 (6.70)		
≥ 3 times a week	322 (93.30)		

### Latent profile analysis of social connectedness among MHD patients

The 9 items of the Social Connectedness Scale were used as manifest variables for latent profile analysis, and models with 1 to 5 latent categories were fitted sequentially, as shown in Table 2. AIC, BIC and aBIC values declined with an increasing number of latent classes. Multiple indicators were comprehensively evaluated to determine the optimal model. ① For the likelihood ratio tests, LMRT (*P* = 0.0119) and BLRT (*P* < 0.001) were significant for the 3-class model, indicating superior model fit relative to the 2-class model. Although LMRT remained significant for the 4-class model (*P* = 0.0126), it became non-significant for the 5-class model (*P* = 0.2155), indicating potential overfitting for models with more than three classes. ② Regarding classification quality, the entropy values were 0.937 for the 2-class model and 0.890 for the 3-class model. The entropy of the 3-class model remained above the conventional cutoff of 0.8, indicating satisfactory classification accuracy. The average posterior probability of correct assignment for the 3-class model ranged from 93.2% to 97.4% (Table 3), further confirming stable and robust classification results. ③ In terms of sample distribution and interpretability, the 3-class model yielded balanced sample proportions of 25.8%, 42.3%, and 31.9%. Each subgroup exhibited distinguishable clinical features with favourable theoretical and practical interpretability. In comparison, the smallest class accounted for only 11.9% in the 4-class model and 9.6% in the 5-class model; subgroups of such small sizes lack practical clinical applicability. ④ Furthermore, the aBIC trend plot showed a marked slowdown in the decline of values at the 3-class point (Supplementary Fig. S1), providing additional evidence for selecting the 3-class solution. Overall, the 3-class latent profile model was identified as the optimal model.

**Table 2:** Comparison of fit indices between models.

Model	AIC	BIC	aBIC	Entropy	LMRT (*P*)	BLRT (*P*)	Category probability
1	10922.082	10991.266	10934.165	-	-	-	1
2	9572.372	9679.991	9591.168	0.937	<0.001	<0.001	0.617/ 0.383
3	9185.834	9331.888	9211.342	0.890	0.0119	<0.001	0.319/ 0.423/0.258
4	9015.637	9200.127	9047.858	0.885	0.0126	<0.001	0.119/0.272/ 0.229/0.380
5	8958.189	9181.115	8997.123	0.871	0.2155	<0.001	0.229/0.096/0.278/0.194/0.203

Note: AIC = Akaike information criterion; BIC = Bayesian information criterion; aBIC = adjusted Bayesian information criterion; LMRT = Lo-Mendell-Rubin likelihood ratio test; BLRT = Bootstrapped Likelihood ratio test.

**Table 3:** Average latent class probabilities for most likely latent class membership (row) by latent class (column).

Class	Profile1	Profile2	Profile3
Profile1	0.955	0.045	0.000
Profile2	0.045	0.932	0.023
Profile3	0.000	0.026	0.974

**Fig. 2:**
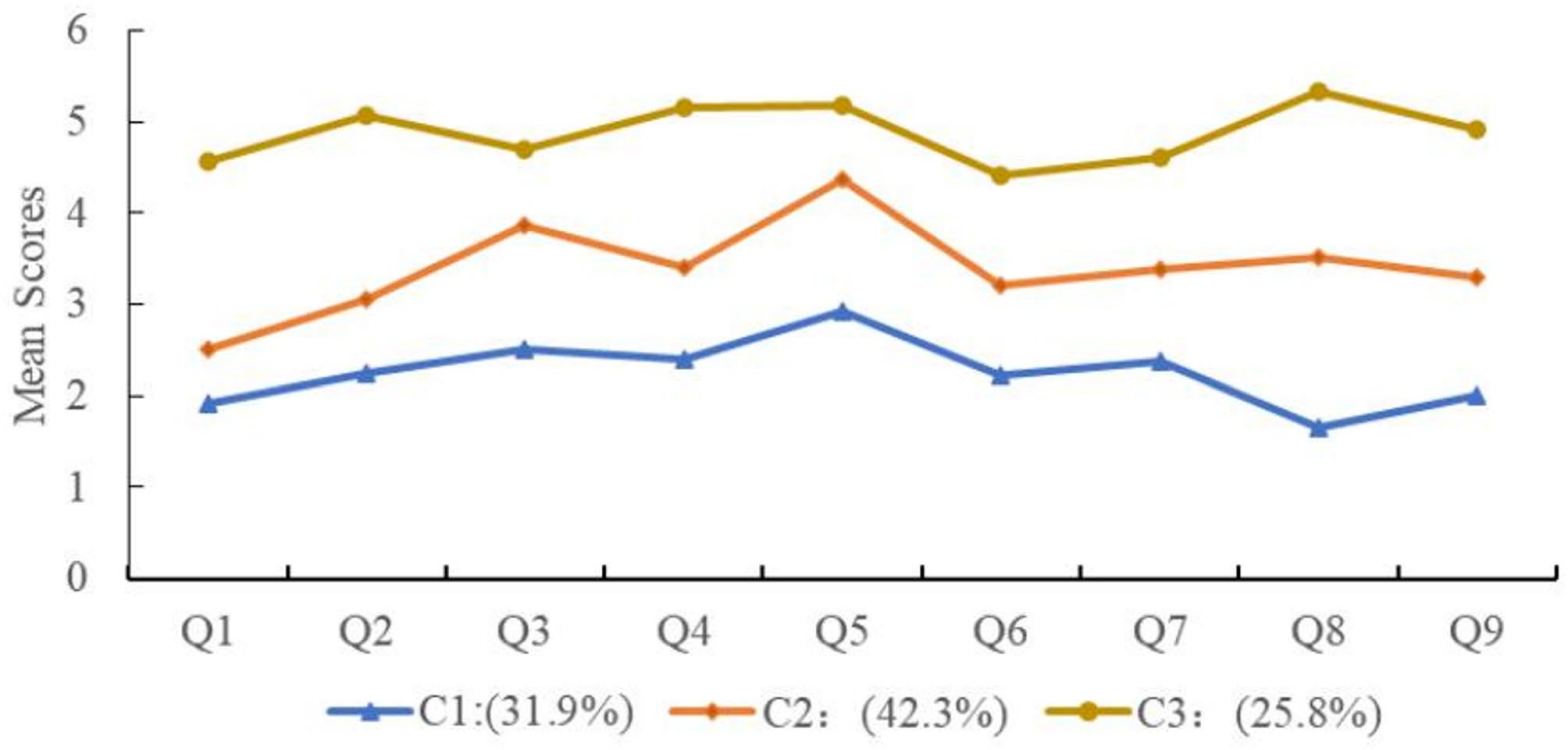
Latent profile model of social connectedness among MHD patients.

The distribution of latent profiles is shown in Fig. 2. The first profile comprised 110 patients (31.9%), with the lowest scores on all 9 items; this group was designated the “low social connectedness group”. Profile 2 had moderate scores between Profile 1 and Profile 3, and was named the “moderate social connectedness group”, comprising 146 cases (42.3%). Profile 3 had the highest scores across all items and was designated the “high social connectedness group”, consisting of 89 patients (25.8%).

### Univariate analysis of latent profiles of social connectedness among MHD patients

The results of the univariate analysis showed that, across the three latent profiles of social connectedness scores among MHD patients (low, moderate, and high groups), educational level, employment status, monthly per capita household income, number of medications, types of chronic diseases, ability to perform activities of daily living, self-rated health status, dialysis shift, psychological resilience, and social support exhibited statistically significant differences across the different social connectedness groups (*P*<0.05). Further details are presented in Table 4.

**Table 4:** Univariate analysis of latent categories of social connectedness in MHD patients.

Variable	C1 (*n* = 110)	C2 (*n* = 146)	C3 (*n* = 89)	Statistics	*P*
Gender [n, (%)]				1.588^a^	0.452
Male	62 (56.40)	92 (63.00)	57 (64.00)		
Female	48 (43.60)	54 (37.70)	32 (36.00)		
Age (years) [n, (%)]				3.408^b^	0.182
18–44	14 (12.70)	16 (11.00)	19 (21.30)		
45–59	30 (27.30)	59 (40.40)	26 (29.20)		
≥ 60	66 (60.00)	71 (48.60)	44 (49.40)		
Marital status [n, (%)]				8.489^a^	0.075
Married	79 (71.80)	122 (83.60)	76 (85.40)		
Unmarried	8 (7.30)	5 (3.40)	5 (5.60)		
Divorced/ Widowed	23(20.90)	19 (13.00)	8 (9.00)		
Place of residence [n, (%)]				3.266^a^	0.195
Rural	28 (25.50)	36 (24.70)	14 (15.70)		
Urban	82 (74.50)	110 (75.30)	75 (84.30)		
Education level [n, (%)]				16.119^b^	< 0.001
Junior high school or lower	53 (48.20)	51 (34.90)	26 (29.20)		
High school or vocational school	41 (37.30)	58 (39.70)	26 (29.20)		
College degree or above	16 (14.5)	37 (25.30)	37 (41.60)		
Employment status [n, (%)]				13.895^a^	0.008
Currently employed	11 (10.00)	30 (20.50)	27 (30.30)		
Leaving employment/ Unemployed	70 (63.60)	83 (56.80)	40 (44.90)		
Retirement	29 (26.40)	33 (22.60)	22 (24.70)		
Monthly per capita household income (yuan) [n, (%)]				19.263^b^	< 0.001
< 3000	59 (53.60)	58 (39.70)	26 (29.20)		
3000–6000	44 (40.00)	63 (43.20)	38 (42.70)		
> 6000	7 (6.40)	25 (17.10)	25 (28.10)		
Medical insurance [n, (%)]				6.269^a^	0.180
Employee medical insurance	39 (35.50)	64 (43.80)	47 (52.80)		
New rural cooperative medical scheme	19 (17.30)	23 (15.80)	10 (11.20)		
Resident medical insurance	52 (47.30)	59 (40.40)	32 (36.00)		
Primary disease [n, (%)]				8.951^a^	0.176
Chronic glomerulonephritis	41 (37.30)	51 (34.90)	32 (36.00)		
Hypertensive nephropathy	19 (17.30)	38 (26.00)	30 (33.70)		
Diabetic nephropathy	32 (29.10)	37 (25.30)	15 (16.90)		
Others	18 (16.40)	20 (13.70)	12 (13.50)		
Number of medications (types) [n, (%)]				26.459^b^	< 0.001
< 3	46 (41.80)	90 (61.60)	69 (77.50)		
≥ 3	64 (58.20)	56 (38.40)	20 (22.50)		
Dialysis frequency [n, (%)]				4.069^b^	0.131
< 3 times a week	3 (2.70)	12 (8.20)	8 (9.00)		
≥ 3 times a week	107 (97.30)	134 (91.80)	81 (91.00)		
Dialysis vintage [n, (%)]				2.702^b^	0.259
< 1 year	8 (7.30)	25 (17.10)	18 (20.20)		
1–5 years	58 (52.70)	63 (43.20)	40 (44.90)		
>5 years	44 (40.00)	58 (39.70)	31 (34.80)		
Types of chronic diseases [n, (%)]				31.855^b^	< 0.001
2	24 (21.80)	66 (45.20)	45 (50.60)		
3	53 (48.20)	71 (48.60)	33 (37.10)		
≥ 4	33 (30.00)	9 (6.20)	11 (12.40)		
Weekly dialysis duration (h) [n, (%)]				0.962^b^	0.618
< 12	11 (10.00)	14 (9.60)	12 (13.50)		
≥ 12	99 (90.00)	132 (90.40)	77 (86.50)		
Activities of daily living [n, (%)]				31.282^a^	< 0.001
Independent	63 (57.30)	120 (82.20)	77 (86.50)		
Partially dependent	36 (32.70)	25 (17.10)	11 (12.40)		
Completely dependent	11 (10.00)	1 (0.70)	1 (1.10)		
Self-rated health status [n, (%)]				11.650^a^	0.003
Poor	43 (39.10)	33 (22.60)	13 (14.60)		
Fair	31 (28.20)	51 (34.90)	34 (38.20)		
Good	36 (32.70)	62 (42.50)	42 (47.20)		
Dialysis shift [n, (%)]				13.573^a^	0.009
Morning dialysis	43 (39.10)	63 (43.20)	41 (46.10)		
Afternoon dialysis	56 (50.90)	73 (50.00)	30 (33.70)		
Evening dialysis	11 (10.00)	10 (6.80)	18 (20.20)		
Scores of CD-RISC-10[M(P_25,_ P_75_)]	16.0 (13.0,21.0)	21.0 (18.0,24.0)	25.0 (22.0,28.0)	80.104^b^	< 0.001
Scores of SSRS[M(P_25_, P_75_)]	27.0 (24.0,30.0)	30.0 (28.0,34.0)	34.0 (31.0,39.0)	75.402^b^	< 0.001

Note: a = chi-square test; b= Kruskal-Wallis *H* test. C1, low social connectedness group; C2, moderate social connectedness group; C3, high social connectedness group. M, Median; P_25_, The 25th percentile; P_75_, The 75th percentile.

### Multivariate analysis of latent profiles of social connectedness among patients with MHD

The multicollinearity test revealed that *VIF* values ranged from 1.094 to 1.513, indicating no multicollinearity among the variables. Using the three latent categories of social connectedness as the dependent variable (low social connectedness group = 1, moderate social connectedness group = 2, high social connectedness group = 3), with the low social connectedness group as the reference group, and employing the items found to be statistically significant in the univariate analysis as independent variables, a multivariate logistic regression analysis was conducted (a parallelism test yielded χ^2^ = 22.423, *P* = 0.033 < 0.05; thus, a multivariate unordered logistic regression analysis was performed). Psychological resilience and social support scores were treated as continuous variables and entered into the model as raw scores; coding for other variables is presented in Table 5. The likelihood ratio chi-square was 205.216 (*P* < 0.001), and the Nagelkerke R² was 0.507, showing adequate overall model fit and good explanatory capacity. Medical insurance type and place of residence showed no significant associations in univariate analysis (*P* > 0.05), and these variables were excluded from the model. Furthermore, sensitivity analysis (Supplementary Table S1) validated this exclusion and confirmed the robustness of our findings. The analysis revealed that lower educational level, retirement status, and having multiple chronic diseases were significantly associated with the “low social connectedness group”. Morning dialysis shift, higher psychological resilience, and higher social support were associated with the “moderate social connectedness group” and the “high social connectedness group” (*P* < 0.05). Detailed results are shown in Table 6.

**Table 5:** Assignment of independent variables.

Independent variable	Assignment method
Education level	Junior high school or lower (1,0), high school or vocational school (0,1), college and above (0,0)
Employment status	Currently employed (1,0), leaving employment/unemployed (0,1), retirement (0,0)
Per capita monthly income	< 3000 (1,0), 3 000-6000 (0,1), > 6000 (0,0)
Number of medications	< 3 (1,0), ≥ 3 (0,1)
Types of chronic diseases	2 (1,0), 3 (0,1), ≥ 4 (0,0)
Activities of daily living	Independent (1,0), partially dependent (0,1), completely dependent (0,0)
Self-rated health status	Poor (1,0), fair (0,1), good (0,0)
Dialysis shift	Morning (1,0), afternoon (0,1), evening (0,0)

**Table 6:** Results of multivariate logistic regression analysis for latent categories of social connectedness among MHD patients.

Item	B	SE	Waldχ^2^	*P*	OR	95%CI
C2 vs. C1						
Constant	-6.470	2.022	10.243	0.001	-	-
Education level						
Junior high school or lower	-0.999	0.507	3.883	0.049	0.368	0.136–0.995
High school or vocational school	-0.851	0.453	3.530	0.060	0.427	0.176–1.037
Employment status						
Leaving employment/ unemployed	-0.697	0.544	1.643	0.200	0.498	0.171–1.446
Retirement	-1.945	0.613	10.078	0.002	0.143	0.043–0.475
Per capita monthly income						
< 3000	-0.584	0.588	0.986	0.321	0.558	0.176–1.767
3000–6000	-0.531	0.545	0.950	0.330	0.588	0.202–1.710
Number of medications	0.100	0.342	0.850	0.770	1.105	0.565–2.160
Types of chronic diseases						
3	-0.102	0.369	0.077	0.782	0.903	0.438–1.862
≥ 4	-2.146	0.524	16.795	< 0.001	0.117	0.042–0.326
Activities of daily living						
Independent	1.929	1.320	2.133	0.144	6.879	0.517–91.501
Partially dependent	1.770	1.313	1.816	0.178	5.872	0.448–77.036
Self-rated health status						
Poor	-0.152	0.415	0.134	0.715	0.859	0.381–1.937
Fair	0.596	0.392	2.317	0.128	1.816	0.842–3.913
Dialysis shift						
Morning	1.544	0.636	5.895	0.015	4.681	1.346–16.275
Afternoon	1.075	0.612	3.090	0.079	2.930	0.884–9.718
Scores of CD-RISC-10	0.102	0.034	8.865	0.003	1.108	1.036–1.185
Scores of SSRS	0.144	0.038	14.067	< 0.001	1.154	1.071–1.244
C3 vs. C1						
Constant	-12.590	2.974	17.924	< 0.001	-	-
Education level						
Junior high school or lower	-1.240	0.596	4.332	0.037	0.289	0.090–0.930
High school or vocational school	-1.291	0.531	5.902	0.015	0.275	0.097–0.779
Employment status						
Leaving employment/ unemployed	-0.953	0.631	2.282	0.131	0.385	0.112–1.328
Retirement	-2.508	0.713	12.387	< 0.001	0.081	0.020–0.329
Per capita monthly income						
< 3000	-0.663	0.674	0.968	0.325	0.515	0.138–1.931
3000–6000	-0.595	0.608	0.956	0.328	0.552	0.167–1.818
Number of medications	0.739	0.452	2.668	0.102	2.093	0.863–5.079
Types of chronic diseases						
3	-0.069	0.461	0.023	0.881	0.933	0.378–2.305
≥ 4	-1.088	0.613	3.152	0.076	0.337	0.101–1.120
Activities of daily living						
Independent	3.054	2.300	1.764	0.184	21.199	0.234-1921.588
Partially dependent	2.978	2.322	1.646	0.200	19.651	0.208-1860.153
Self-rated health status						
Poor	-0.400	0.556	0.517	0.472	0.670	0.225–1.994
Fair	0.663	0.476	1.942	0.163	1.941	0.764–4.932
Dialysis shift						
Morning	0.851	0.693	1.508	0.219	2.342	0.602–9.105
Afternoon	-0.308	0.674	0.209	0.648	0.735	0.196–2.754
Scores of CD-RISC-10	0.222	0.045	24.883	< 0.001	1.249	1.144–1.363
Scores of SSRS	0.226	0.045	25.670	< 0.001	1.254	1.149–1.369

Note: C1, low social connectedness group; C2, moderate social connectedness group; C3, high social connectedness group.

## Discussion

### Significant heterogeneity was observed in social connectedness among MHD patients

In this study, the social connectedness score among MHD patients was 30.77 ± 9.96, indicating a moderate level of social connectedness and room for overall improvement. Patients with MHD endure repeated disease-related distress over the long term. The cumulative impact of physical suffering and psychological stress gradually impairs their ability to form connections with others^5^. Furthermore, the unemployment rate among MHD patients in this study was as high as 55.90%. Their deteriorating financial circumstances led to a decline in quality of life, and patients with poor quality of life exhibited poorer social reintegration with regard to social participation^33,34^. These findings indicate that healthcare professionals should pay close attention to this issue and develop targeted interventions to effectively enhance patients’ levels of social connectedness.

The results of this study indicate that patients with MHD can be categorised into three groups based on their social connectedness: low, moderate and high. There is significant heterogeneity among these groups. The high social connectedness group accounted for 25.8%; members of this group exhibited a high level of social connectedness, with good psychological resilience and strong social support, enabling them to actively integrate into their surroundings. The moderate social connectedness group (42.3%) constituted the largest cohort; these patients scored highly on item 5 (I feel isolated from the world around me), suggesting that a sense of social isolation is prevalent in this group. This may be related to the high proportion of patients undergoing long-term dialysis in this category. Long-term, high-frequency dialysis treatment consumes a significant amount of time and energy that could otherwise be devoted to socialising. Furthermore, physical fatigue and altered self-perception resulting from treatment further diminish their willingness to actively establish and maintain social connections. The low social connectedness group (31.9%) was the second largest. These patients scored low on item 8 (I find myself disconnected from society), reflecting a strong sense of belonging deficit. They face dual pressures from financial burden and health concerns, and have a high risk of depression, which may exacerbate their social disengagement. This suggests that this group is in greatest need of psychological and social support.

Therefore, for healthcare professionals working with the high social connectedness group, the approach should focus on support and encouragement, making full use of these patients’ strengths and encouraging them to assist other patient groups in improving their social connectedness through role modelling and the sharing of experiences. For the moderate social connectedness group, the emphasis should be on addressing social needs; healthcare professionals need to focus on helping these patients build healthy relationships, for example by organising peer support activities, strengthening guidance on family involvement, and improving dynamics between patients and their families. For the low social connectedness group, healthcare professionals must identify issues at an early stage and intervene immediately. Multiple studies have confirmed that cognitive-behavioral therapy (CBT) alleviates negative emotions and social avoidance among MHD patients^35,36^. Nevertheless, traditional face-to-face CBT poses implementation challenges. Digital CBT based on intelligent audio applications^37^ or one-on-one individualized CBT during dialysis sessions is therefore recommended. Combined with psychological counselling and medical support, patients can restructure their cognition and prevent a further decline in social connectedness.

### Factors associated with social connectedness in MHD patients

#### Education level

Our findings revealed that lower educational attainment was associated with belonging to the low social connectedness group among MHD patients. Specifically, compared with those with a college degree or higher, patients with junior high school or lower education were significantly less likely to be classified into the moderate (OR = 0.368, *P* = 0.049) or high (OR = 0.289, *P* = 0.037) social connectedness groups, and those with high school or vocational school education were also significantly less likely to be classified into the high social connectedness group (OR = 0.275, *P* = 0.015), consistent with previous findings^38^. Patients with lower educational attainment often have lower health literacy and limited skills to express personal feelings, which may coincide with avoidant and denial coping patterns during illness, as well as reduced social engagement and poorer confidence in social reintegration^39^. Restricted emotional expression is also associated with poor self-regulation and multiple psychological issues^40^. By contrast, patients with higher educational attainment possess stronger skills in information acquisition and cognitive adjustment^41^, which help relieve stress related to illness uncertainty and mitigate social withdrawal. Consequently, for patients with lower educational attainment, healthcare professionals may use visual materials such as images and videos to enhance health education; provide psychological counselling to guide patients in expressing their inner feelings, thereby improving disease understanding and their social confidence. Meanwhile, encouraging patients to engage in more social interactions, assisting them in establishing a robust interpersonal support network, and improving their mental health can further promote their overall level of social connectedness.

#### Employment status

Compared with employed counterparts, retired MHD patients were far less likely to be categorized into the moderate or high social connectedness groups (OR = 0.143, *P* = 0.002; OR = 0.081, *P* < 0.001), indicating a significant association between retirement and the low social connectedness group. This finding contrasts with the results reported by Wang et al.^42^. This discrepancy may coincide with the loss of original workplace social networks following retirement, alongside a gradual reduction in social opportunities. Secondly, retired MHD patients show a gradual decline in physiological function with advancing age. They are often accompanied by complications such as frailty, systemic inflammation, and cognitive decline^3^, which may restrict their participation in social activities. Consequently, patients tend to reduce social interactions voluntarily or involuntarily, have less contact with the outside world, and face a higher risk of social disconnection. This suggests that healthcare professionals should pay close attention to retired MHD patients. Alongside disease treatment and complication management, interest-based physical activities can be organized based on individual physical capacity. Moderate exercise can significantly improve physiological function, frailty status and quality of life among MHD patients, and its benefits in this group have been extensively validated^43,44^. We recommend prioritizing small-scale, short-duration activities such as modified seated Baduanjin^45^. Community resources and WeChat group check-ins can also be used to establish community interest groups, encouraging patients with similar physical conditions to take part in mild-to-moderate exercise together at home or in local communities^46^. These activities can not only improve physical function and motivate patients to stay active, but also help them gradually rebuild social routines.

#### Dialysis shift

The results of this study indicate that, compared with evening dialysis, MHD patients undergoing dialysis in the morning are more likely to be classified as having moderate social connectedness (OR = 4.681, *P* = 0.015). Mornings typically correspond to peak energy levels and relatively high cortisol concentrations^47^; patients receiving morning dialysis may experience milder post-dialysis fatigue, which may coincide with better preserved social functioning. In addition, patients receiving morning dialysis have the whole afternoon and evening available for social activities, family gatherings and leisure pursuits, which generally creates more opportunities for social connectedness. Therefore, medical staff may optimise dialysis scheduling when ward conditions permit. For patients undergoing evening dialysis, targeted guidance can be provided to their family members to offer more evening companionship and regular telephone contact, so as to make up for insufficient social interaction and help maintain their social connectedness.

### Types of chronic diseases

The present study revealed that MHD patients with four or more chronic conditions were significantly less likely to be classified into the moderate social connectedness group (OR = 0.117, *P* < 0.001), suggesting that patients with more chronic comorbidities tend to fall into the low social connectedness group, which is consistent with the findings of Cudjoe et al.^48^. Multiple chronic conditions may interact and exert cumulative effects, raising the burden of disease management such as blood glucose monitoring and medication adjustments. These tasks not only deplete patients’ energy but may also coincide with negative emotions such as anxiety and helplessness, which may weaken their sense of control over social interactions and related expectations. Those with fewer comorbidities appeared to have better psychological wellbeing. MHD patients, however, commonly have concurrent cardiovascular disease, diabetes and other illnesses^48^. These findings underscore the importance of assessing the number and severity of patients’ chronic conditions and suggest that optimising comorbidity management strategies may help alleviate the burden of disease. Continuous care delivered via internet platforms and remote guidance^49^ could help reduce geographical barriers and potentially facilitate sustained social engagement, stronger recovery confidence, and improved self-management capacity.

### **Psychological resilience score**

The findings of this study indicate that, compared with the low social connectedness group, patients with higher psychological resilience scores were more likely to be classified into the moderate and high social connectedness groups (OR = 1.108, *P* = 0.003; OR = 1.249, *P* < 0.001). Psychological resilience is positively associated with social connectedness, which is consistent with previous research findings^50^. While the effect size of psychological resilience for one category was relatively small (OR = 1.108), this association still carries clinical implications. Individuals with high psychological resilience generally have greater resilience to stress^17^. It is plausible that higher resilience coincides with milder dialysis-related stress and a greater tendency to adopt positive coping strategies, such as actively seeking social support, using available resources and engaging in social activities. Regular screening for psychological resilience may be considered. Mindfulness-based stress reduction and relaxation training are well-documented approaches, and prior interventional studies suggest they correlate with favourable psychological status and elevated resilience among dialysis patients^51,52^. Existing resources within the department can be utilised to deliver these approaches during fragmented periods, such as while patients are waiting for or receiving dialysis. For example, patients can receive audio-guided mindfulness meditation and progressive muscle relaxation during hemodialysis sessions. This approach fully utilises patients’ downtime during treatment, does not substantially increase staff workload, is low-cost, and could be readily integrated into routine dialysis care.

### Social Support Score

The findings of this study indicate that, compared with the low social connectedness group, MHD patients with higher levels of social support are more likely to be classified into the moderate and high social connectedness groups (OR = 1.154, *P <* 0.001; OR = 1.254, *P* < 0.001), consistent with previous research findings^53^. Higher levels of social support are often accompanied by greater care and companionship that patients receive from family, friends and other sources^54^, and feeling respected in social settings. Such support may be associated with greater engagement in social activities and better social integration. The convoy model^55^ posits that individuals sustain intimate bonds via stable family, friend and other supportive networks, characteristics correlated with lower perceived stress and greater adaptive capacity. Based on these findings, it may be useful for clinicians to mobilise patients’ social resources to build a multi-dimensional support system. Family members and peers could be encouraged to provide support through online communication and in-person company. When delivering supportive approaches, clinical staff might consider fully respecting patients’ needs and feedback and adjusting care strategies dynamically.

Notably, this study adopted the social-ecological systems theory to identify potential associated factors across micro, meso and macro levels. Micro- and meso-level factors were significantly linked to the subgroup distribution of patients’ social connectedness, while macro indicators, including residential location and medical insurance, yielded no statistically significant differences. We speculate that this phenomenon is related to sample characteristics, rather than implying a lack of correlations between the macro environment and social connectedness. Dialysis-related medical insurance policies are widely accessible in China, and nearly all MHD patients in this study received stable medical coverage. As a universal protective factor, macro-level policies showed no relationship with between-group disparities. In addition, urban patients made up 77.4% of the sample, a feature accompanied by high geographical homogeneity and reduced discriminative ability of the residential location variable.

These results suggest that among MHD patients with sound medical insurance and limited regional diversity, differences in social connectedness are mainly affected by micro- and meso-level factors. Future research could expand the sample scope and conduct cross-regional and urban-rural comparative studies to further explore the roles of macro factors and enrich the application of social-ecological systems theory in this field.

### Limitations

This study has several limitations. Firstly, the study employed convenience sampling and participants were limited to Anhui Province, which may limit the generalizability of the findings to other sociocultural contexts, particularly to regions with substantially different healthcare resources or socioeconomic conditions. Secondly, as this was a cross-sectional study, it cannot establish causal relationships. Finally, all measurements relied on patients’ self-reports, which may be subject to reporting bias. Furthermore, due to the small sample sizes in some subgroups, the confidence intervals for the parameter estimates were wide, limiting precision and potentially affecting the stability of the results.

These limitations suggest that the results should be interpreted with caution and highlight the need for external validation. Consequently, future research could adopt random or stratified sampling to improve sample representativeness, expand the study scope and recruitment sources, and conduct multi-centre longitudinal studies. Integrating objective clinical indicators into comprehensive analyses would further deepen the research.

Despite these limitations, this study innovatively combined latent profile analysis with social-ecological systems theory to systematically reveal the heterogeneous characteristics and key factors of social connectedness among MHD patients in China. This novel approach extends beyond traditional variable-centred analyses and provides new insights for clinical practice. By identifying distinct patient subgroups, our findings highlight the need for targeted interventions.

## Conclusion

Patients undergoing maintenance hemodialysis can be categorised into groups with low, moderate, and high levels of social connectedness, demonstrating significant heterogeneity across these groups. Those with lower educational attainment, who are retired, or who have more chronic conditions are more likely to be classified in the low social connectedness group, whereas patients undergoing dialysis in the morning, those with good psychological resilience, and those with high levels of social support are more likely to be classified in the moderate and high social connectedness groups. In clinical practice, healthcare professionals should identify individual differences among patients based on the characteristics of their social connectedness. By implementing stratified management interventions, they can facilitate patients’ better integration into society, thereby enhancing their overall psychosocial adaptive capacity.

## Supplementary Information

Below is the link to the electronic supplementary material.

Supplementary Material 1

## Data Availability

The data used and analysed in this study may be obtained from the corresponding author upon reasonable request.
