# Supplementary material for: Latent profiles of social connectedness and associated factors in maintenance hemodialysis patients
Source: Sci Rep. 2026 Jun 24;16:25883. doi: 10.1038/s41598-026-59651-z (PMC13487171; doi:10.1038/s41598-026-59651-z)
Supplement: Supplementary file 1 [file 41598_2026_59651_MOESM1_ESM.pdf]

Latent profiles of social connectedness and associated factors in maintenance hemodialysis patients

Yun Zhang[1], Zhiyan Sun[1], Naiyue Ye[2], Xun Zhou[3], Menghan Zheng[3], Peili Xu*[4]

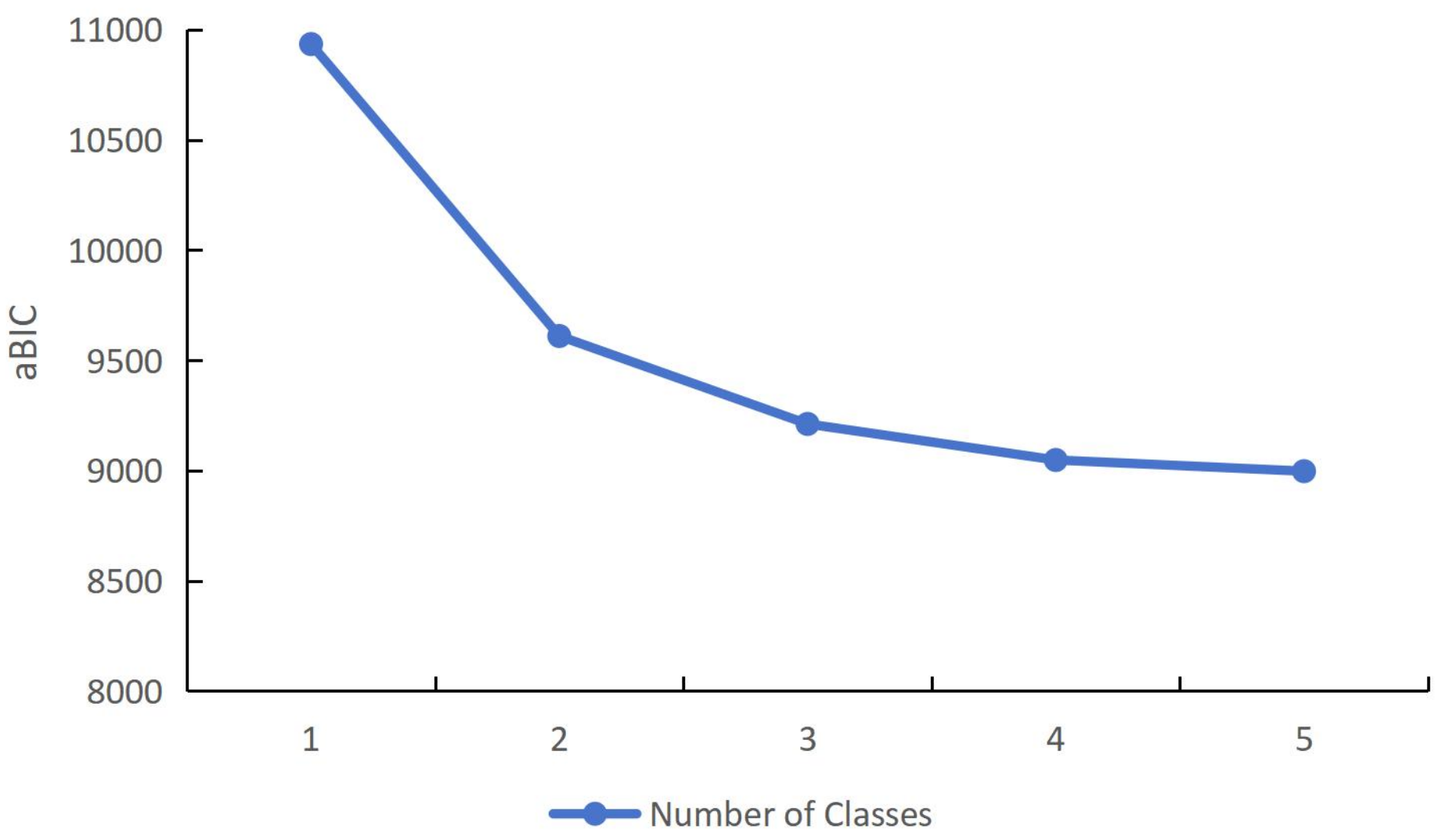

Supplementary Fig. S1. Trend plot.

Table S1.Sensitivity analysis results

Item	B	SE	Waldχ^2	*P*	*OR*	95%*CI*
**C2 vs. C1**						
Constant	-6.670	2.102	10.071	0.002	–	–
Education level						
Junior high school or lower	-1.059	0.532	3.954	0.047	0.347	0.122–0.985
High school or vocational school	-0.899	0.463	3.774	0.052	0.407	0.164–1.008
**Residence**						
**Rural**	**-0.001**	**0.416**	**< 0.001**	**0.999**	**0.999**	**0.442–2.259**
Employment status						
Leaving employment/unemployed	-0.640	0.570	1.261	0.261	0.527	0.173–1.611
Retirement	-1.966	0.646	9.256	0.002	0.140	0.039–0.497
Per capita monthly income						
< 3000	-0.577	0.588	0.962	0.327	0.562	0.177–1.779
3000-6000	-0.504	0.545	0.854	0.355	0.604	0.208–1.759
**Medical insurance**						

**Employee medical insurance**	**0.136**	**0.496**	**0.075**	**0.784**	**1.146**	**0.434−3.027**
**New Rural Cooperative Medical Scheme**	**0.288**	**0.480**	**0.361**	**0.548**	**1.334**	**0.521−3.416**
Number of medications	0.091	0.344	0.070	0.792	1.095	0.558−2.147
Types of chronic diseases						
3	-0.113	0.377	0.090	0.764	0.893	0.427−1.869
⩾4	-2.128	0.528	16.271	< 0.001	0.119	0.042−0.335
Activities of daily living						
Independent	1.996	1.341	2.217	0.137	7.360	0.532−101.852
partially dependent	1.833	1.331	1.898	0.168	6.255	0.461−84.918
Self-rated health status						
Poor	-0.125	0.417	0.090	0.764	0.882	0.390−1.997
Fair	0.593	0.392	2.286	0.131	1.809	0.839−3.900
Dialysis shift						
Morning	1.533	0.644	5.670	0.017	4.632	1.312−16.360
Afternoon	1.056	0.621	2.896	0.089	2.875	0.852−9.701
Scores of CD-RISC-10	0.106	0.035	9.220	0.002	1.111	1.038−1.190
Scores of SSRS	0.143	0.039	13.666	< 0.001	1.154	1.070−1.245
C3 vs. C1						
Constant	-12.482	3.166	15.546	< 0.001	−	−
Education level						
Junior high school or lower	-1.348	0.629	4.598	0.032	0.260	0.076−0.891
High school or vocational school	-1.432	0.547	6.847	0.009	0.239	0.082−0.698
Employment status						
**Residence**						
**Rural**	**-0.452**	**0.543**	**0.694**	**0.405**	**0.636**	**0.219−1.844**
Leaving employment/unemployed	-1.041	0.667	2.438	0.118	0.353	0.096−1.304
Retirement	-2.456	0.753	10.626	0.001	0.086	0.020−0.376
Per capita monthly income						
< 3000	-0.643	0.680	0.893	0.345	0.526	0.139−1.995
3000-6000	-0.524	0.611	0.737	0.391	0.592	0.179−1.959
Number of medications	0.701	0.460	2.323	0.127	2.016	0.818−4.968
**Medical insurance**						
**Employee medical insurance**	**-0.192**	**0.616**	**0.097**	**0.755**	**0.825**	**0.274−2.760**
**New Rural Cooperative Medical Scheme**	**0.739**	**0.614**	**1.451**	**0.228**	**2.094**	**0.629−6.972**
Types of chronic diseases						
3	-0.167	0.469	0.127	0.721	0.846	0.337−2.121

⩾ 4	-1.109	0.619	3.215	0.073	0.330	0.098−1.109
Activities of daily living						
Independent	2.924	2.468	1.404	0.236	18.614	0.148−2347.759
partially dependent	2.862	2.489	1.322	0.250	17.490	0.133−2296.588
Self-rated health status						
Poor	-0.361	0.561	0.413	0.520	0.697	0.232−2.095
fair	0.707	0.480	2.169	0.141	2.028	0.792−5.196
Dialysis shift						
Morning	0.875	0.702	1.544	0.212	2.398	0.606−9.488
Afternoon	-0.303	0.685	0.195	0.659	0.739	0.193−2.829
Scores of CD-RISC-10	0.226	0.045	25.082	< 0.001	1.253	1.147−1.369
Scores of SSRS	0.231	0.046	25.748	< 0.001	1.260	1.153−1.378

Note: Some subgroups of the Activities of daily living variable (Independent / partially dependent) show extremely wide confidence intervals, coinciding with very small sample sizes within these subgroups that correspond to elevated standard error and reduced estimation precision. These results should be interpreted with caution.
